# Number of Natural Teeth and Oral Impacts: A Study on Sri Lankan Adults

**DOI:** 10.1155/2011/809620

**Published:** 2011-11-16

**Authors:** Roshnal Perera, Lilani Ekanayake

**Affiliations:** ^1^School of Dentistry and Oral Health, Griffith University, Gold Coast Campus, Gold Coast, QLD 4215, Australia; ^2^Department of Community Dental Health, Faculty of Dental Science, University of Peradeniya, Peradeniya 20400, Sri Lanka

## Abstract

The aim of the study was to determine the association between the number of natural teeth and oral impacts in Sri Lankan adults. The sample consisted of 476, 40–59 and 452, ≥60 year olds. Oral impacts were assessed using a validated Sinhalese translation of the Oral Health Impact Profile-14 scale. A receiver-operating characteristic (ROC) curve was plotted to determine the number of natural teeth that would best discriminate those with oral impacts from those without. Oral impacts were reported by 26% of the 40–59 year olds and 34% of the older individuals. In both groups there was a significant negative correlation between the number of teeth present and oral impacts. The ROC curve for the 40–59 year olds gave an area under the curve (AUC) of 0.758 (95% CI = 0.702–0.814; *P* < 0.001) with an optimal cut-off of 24/25 teeth while for the ≥60 year olds, the AUC of the ROC curve was 0.737 (95% CI = 0.684–0.790; *P* < 0.001) with an optimal cut-off of 18/19 teeth. Based on the ROC curves the optimal cutoffs of the number of natural teeth that best discriminated between those with and without oral impacts for 40–59 and ≥60 year olds were 24-25 and 18-19, respectively.

## 1. Introduction

There has been a considerable interest in the assessment of subjective impacts of oral disorders on the quality of life in recent times. Several instruments have been developed for this purpose, and they assess impairments associated with physical, social, and psychological well-being. The relationships between various oral health-related variables and oral impacts have been explored, and there is unequivocal evidence to suggest that the number of natural teeth is one of the key factors associated with oral impairment [1, 2]. 

The number of teeth needed for satisfactory performance of various oral functions has been the topic of many studies. The World Health Organization identified the maintenance of a natural dentition of not less than 20 teeth throughout life as one of the global indicators for the year 2000 [3]. A systematic review which was conducted over two decades later to evaluate the relationship between the dentition and oral function has also reported that a dentition consisting of 20 teeth would assure an acceptable level of oral function [4]. However, Steele et al. [5], based on the findings of their study on the effect of tooth loss on oral health impacts and quality of life conducted among two national samples from England and Australia, argued that the threshold of 20-21 teeth for a functional dentition would never be universally applicable. Considering the above argument it would be of interest to determine how tooth loss could impact the oral-health-related quality of life of Sri Lankan adults who are socioculturally different from those populations from the developed countries or whether the threshold of 20 teeth for an acceptable level of oral function is applicable to Sri Lankans. Therefore, the aim of this study was to determine the association between the number of natural teeth and oral impacts among Sri Lankan adults.

## 2. Material and Methods 

The data for the present paper was obtained from a broader study that was carried out to assess tooth loss and its effects on the well-being of an adult population aged 20 years and above residing in the Colombo district. Those living in business premises, prisons, hostels, and religious institutions as well as those who were physically and mentally challenged were excluded. Ethical clearance for the study was obtained from the Ethical Review Committee of the Faculty of Medicine, University of Colombo. Also written informed consent was obtained from all participants. The methodology to determine the association between teeth present and oral impacts will be described here. 

Two age groups were considered (40–59 and ≥60 year olds), and the sample size for each age group was calculated separately. The sample size was determined using the formula for estimating a population proportion with absolute precision, and the prevalence rates of tooth loss reported in the National Oral Health survey [6] for 35–44 (80%) and 65–74 year olds (90%) were used for this purpose. Considering the above prevalence rates, a confidence interval of 95% and a margin of error of 5% and 4% for the 40–59-year-old and ≥60-year-old groups, the sample size required for the two groups were 246 and 216, respectively. As a cluster sampling technique was used to select the sample, it was necessary to make allowance for the design effect which was considered as 1.5. Therefore, after adjusting for the design effect and the nonrespondents (20% for the 40–59; 40% for ≥60 year olds), the sizes of the samples required for the two groups were 443 and 454. When a cluster sampling method is used, in order to obtain valid data at least 30 clusters have to be included in a study [7]. As the study population is large and distributed over a wide geographical area, it was decided to select the subjects from 60 clusters to ensure validity. Hence, the sample size derived for each age group was increased to the nearest multiple of 60 which needed 480 subjects per age group and 7 subjects (480/60) per cluster per age group.

 Administration of health services in the Colombo district is carried out by two authorities: the Ministry of Health and the Colombo Municipal Council (CMC). The regions under the purview of these two authorities are further divided into Public Health Inspector (PHI) areas. The 60 clusters were allocated to the two regions based on the population proportions: 17 to the CMC area and 43 to the rest of the district. A PHI area was considered as a cluster unit, and the required number of clusters was identified from the two regions based on the probability proportionate to size technique. Then individuals who satisfied the inclusion criteria were selected by visiting households in each cluster. Only one person from a given age category was selected from a household. The first author collected the data by means of a pretested interviewer administered questionnaire and an oral examination. The questionnaire was used to obtain information on sociodemographic data and oral health behaviours. It also included the Sinhalese translation of the Oral Health Impact Profile-14 (OHIP-14) scale [8] which had been validated previously [9]. The OHIP-14 consists of 14 items about impacts that could arise as a result of problems in teeth, mouth, or dentures, and the respondents are requested to indicate the frequency of experiencing each impact over the past 12 months on a 5-point Likert-type scale: 0 = never, 1 = hardly ever, 2 = occasionally, 3 = fairly often, and 4 = often. However, to minimize recall bias a period of 6 months was considered in the present study. The oral examination was carried out while the subject was seated on an ordinary chair under natural light. The number of teeth present was noted.

SPSS 13.0 software was used for data analysis. The OHIP score for an individual was determined by summing the coded responses for each of the 14 items of the OHIP scale. This measure takes into account impacts experienced at all levels of frequency. The OHIP score for an individual would range from 0 to 56. As the number of teeth present and OHIP scores were not normally distributed, nonparametric tests were used in the data analysis. 

A receiver-operating characteristic (ROC) curve was plotted to determine the number of natural teeth that would best discriminate those with oral impacts from those without. A ROC curve is obtained by calculating the sensitivity and specificity at every possible cut-off point of the continuous predictor variable (test) and plotting sensitivity (rate of true positives) against 1-specificity (rate of false positives). The optimal cutoff at which the predictor variable discriminates between those with and without the outcome of interest is determined by selecting the best compromise between sensitivity and 1-specificity. This cut-off point is indicated by the point on the curve that is closest to the top of the *y*-axis (0, 1 point). To plot the ROC curve, the number of natural teeth present was used as the continuous predictor variable, while the binary outcome variable defining those with and without oral impacts (gold standard) was determined as follows: those reporting one or more of the 14 impacts fairly often or very often (scores 3 or 4) were regarded as having oral impacts, while those who did not report any one of the impacts fairly often or very often were regarded as not having oral impacts. Considering scores 3 and 4 would identify only those whose oral impacts were chronic rather than transitory. Slade [8] who developed the OHIP scale has recommended this method of analysis, and in fact he and his coworkers have used the same in their studies [10]. The primary statistic obtained from ROC analysis is the area under the curve (AUC) which quantifies the overall ability of the continuous predictor variable to discriminate between those with and without the outcome of interest. A perfect predictor would have an AUC of 1.00, while 0.5 represents a useless predictor (when the curve lies on the diagonal line).

## 3. Results

A total of 480 from each age group were selected to be included in the sample. However, only 476 of the 40–59 year olds and 452 of the ≥60 year olds agreed to participate in the study giving a response rate of 97%. Denture wearers were excluded from the analysis, and the results are therefore based on 405 and 379 nondenture wearers in the 40–59 and ≥60 year olds, respectively. Of the nondenture wearers, 23, 81, and 4% had received up to 5, 6–12, and >12 years of education.

**Figure 1:**
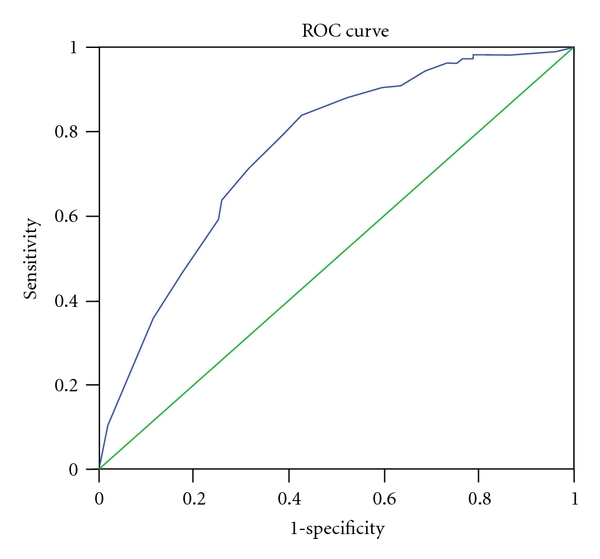
ROC curve for number of natural teeth discriminating those with/without oral impacts in 40–59 year olds. AUC = 0.758 (95% CI = 0.702–0.814); *P* < 0.001. Optimal cutoff based on curve = 24/25 teeth.

**Figure 2:**
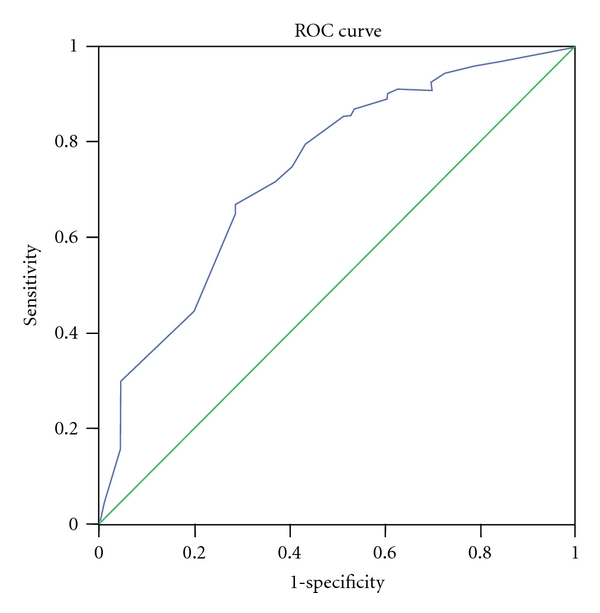
ROC curve for number of natural teeth discriminating those with/without oral impacts in ≥60 year olds. AUC = 0.737 (95% CI = 0.684–0.790); *P* < 0.001. Optimal cutoff based on curve = 18/19 teeth.

The mean and the median number of teeth present in 40–59 year olds were 24.3 and 27, respectively, while, in the ≥60 year olds, the figures for the same variable were 17.4 and 20, respectively. Based on the definition used, oral impacts were reported by 26% of the 40–59 year olds and 34% of the older individuals. In both age groups there was a significant negative correlation between the number of teeth present and OHIP-14 scores. Also, in both age groups, the number of teeth present in those without impacts was significantly higher than in those with impacts (Table 1). Figures 1 and 2 show the ROC curves for 40–59 and ≥60 year olds. For Figure 1, the AUC is 0.758 (95% CI = 0.702–0.814; *P* < 0.001) and the optimal cutoff based on the curve is 24/25 teeth, while for Figure 2 the AUC is 0.737 (95% CI = 0.684–0.790; *P* < 0.001) and the optimal cutoff based on the curve is 18/19 teeth. The diagnostic performance of the cut-off 24/25 natural teeth in detecting those with/without oral impacts in 40–59 year olds is shown in Table 2. Sensitivity and specificity were 0.69 and 0.70, respectively, while the positive predictive value (PPV) was 0.87 and the negative predictive value (NPV) was 0.44.  Table 3 shows the diagnostic performance of the cut-off 18/19 natural teeth in detecting those with/without impacts in ≥60 year olds. Sensitivity and specificity were 0.67 and 0.71, respectively, while the PPV was 0.82 and the NPV was 0.53.

## 4. Discussion

Conforming to the other studies [1, 2, 5], there was a negative association between the number of teeth present and oral impacts. Having observed this association, a ROC analysis was used to determine the number of teeth that would best discriminate those with oral impacts from those without oral impacts.

In medicine, ROC analysis is used to select the optimal cut-off value for a test result, to assess the diagnostic accuracy of a test, and to compare usefulness of different tests [11]. It has been used to predict caries [12] and to determine compliance with oral hygiene [13] in population-based oral health studies. As the predictor variable (number of teeth present) in the present study was measured on a continuous scale, ROC analysis is appropriate to determine the number of teeth that would discriminate those with oral impacts from those without. As denture wear has a positive influence on oral-health-related quality of life of Sri Lankans, denture wearers were excluded from the analysis [14]. Moreover, as tooth loss increases with age and age has an effect on reporting oral impacts [5], it was decided to plot the ROC curves for 40–59 and ≥60 year olds separately. 

The AUC of the ROC analyses which is a measure of accuracy of the predictor were 0.758 and 0.737 for the 40–59- and ≥60-year-old groups, respectively, and statistically significant. *P* values less than 0.001 indicate that the number of teeth does discriminate those with oral impacts from those without oral impacts. According to Fischer et al. [15], the accuracy of a test with an AUC between 0.70 and 0.90 is moderate. This indicates that the parameter number of natural teeth in the mouth was moderately accurate in discriminating those with and with oral impacts. Based on the ROC curves, the optimal cutoffs of teeth that best discriminated between those with and without oral impacts for the 40–59 and ≥60 year olds were 24-25 and 18-19, respectively. 

Several studies have attempted at assessing the number of teeth needed to satisfy functional and social demands. Early studies have attempted at determining the number of teeth needed to satisfy various oral functions such as eating and communication [16, 17], while the more recent studies have assessed the number of teeth below which oral impacts are likely to occur [5]. Hence, meaningful comparisons with previous studies are difficult. Nevertheless, in their study on Brazilian 35–54-year-old male manual workers, Elias and Sheiham [17] found that the probability of satisfaction with the mouth increased with the increase in the number of teeth until 23 teeth and that an increase beyond 23 teeth had no effect on the satisfaction. Having controlled for age, the worst OHIP-14 scores were found when there were less than 21 natural teeth in Australians, while, for adults from the UK, the corresponding figure was fewer than 17 teeth. Moreover, in both populations, those with 25 or more natural teeth had better oral-health-related quality of life than those with less than 25 teeth [5]. The findings for the 40–59 year olds of the present study are in conformity with the above findings. Ueno et al. [18] in their study on 40–75 year olds assessed the number of natural teeth needed to chew 15 food items commonly included in the Japanese diet. They found that having an average of 23.3 natural teeth would allow subjects to eat all 15 food items and those who had problems in eating one or more of the food items had an average of 17.2 teeth. Sheiham et al. [19] found that free-living dentate individuals over the age of 65 years with less than 11 teeth were more likely to have an oral impact than those with more than 11 teeth and is much lower than the figure found for the over 60 year olds of the present study. Methodological variations such as differences in the instruments used to record oral impacts, age groups considered, and the methods of analysis used to calculate the minimum number of teeth may have contributed to the observed differences between studies to a certain extent. Moreover, perception of oral impacts is influenced by cultural norms [5] and hence cultural differences between populations may have been partly responsible for the observed variations in health between studies. 

ROC analysis determines how accurately a continuous predictor variable discriminates between those with/without a condition as defined by the gold standard. The accuracy of ROC analysis depends on the quality of the gold standard considered [11]. There is no gold standard to measure oral impacts, and, in the absence of the ideal, there was no choice but to use one of the instruments such as the OHIP-14 which had been developed to assess impacts associated with oral disorders as the gold standard. The present study assessed the association between the number of teeth and oral impacts. However, it has been shown that the reporting of impacts is influenced not only by the teeth present but also by the condition and position of teeth such as the numbers of occluding posterior and anterior pairs [1]. Therefore, it is recommended that further research is conducted to determine the numbers of occluding pairs of anterior and posterior teeth below which oral impacts are likely to occur.

In conclusion, the results showed that the number of teeth is negatively associated with oral impacts and that the number of teeth that would best discriminate those with and without oral impacts differed according to age group. This indicates that the retention of a dentition of 20 teeth is not necessary for adults of all ages.

## Figures and Tables

**Table 1:** Teeth present and oral impacts in the sample.

	40–59 year olds (*n* = 405)	≥60 year olds (*n* = 379)
Mean (SD) no. of teeth present	24.3 ± 6.8	17.4 ± 9.8
Median no. of teeth present	27	20
Mean (SD) decayed teeth	1.30 ± 1.8	1.12 ± 2.0
Median no. of decayed teeth	0	0

Mean (SD) OHIP score	6.64 ± 6.8	10.50 ± 8.3
Median OHIP score	5	10
% (*n*) with oral impacts	25.7% (104)	34.0% (129)
% (*n*) without oral impacts	74.3% (301)	66.0% (250)
Association between number of teeth and OHIP scores	*r* = −0.496 *P* < 0.001*	*r* = −0.535 *P* < 0.001*

Median no. of teeth		
In those with impacts	21	12
In those without impacts	27 *P* < 0.001**	22 *P* < 0.001**

*Spearman rank correlation;

**Mann-Whitney test.

**Table 2:** Diagnostic performance of 24/25 natural teeth in detecting those with/without oral impacts in 40–59 year olds.

No. of teeth	Oral impacts			Sn	Sp	PPV	NPV
Cutoff	With	Without	Total				
≤24	73	93	166	0.69	0.70	0.87	0.44
≥25	31	208	239				
Total	104	301	405				

Sn: sensitivity; Sp: specificity; PPV: positive predictive value; NPV: negative predictive value.

**Table 3:** Diagnostic performance of 18/19 natural teeth in detecting those with/without oral impacts in ≥60 year olds.

No. of teeth	Oral impacts			Sn	Sp	PPV	NPV
Cutoff	With	Without	Total				
≤18	92	83	175	0.67	0.71	0.82	0.53
≥19	37	167	204				
Total	129	250	379				

Sn: sensitivity; Sp: specificity; PPV: positive predictive value; NPV: negative predictive value.
